# On the In Vitro and In Vivo Hazard Assessment of a Novel Nanomaterial to Reduce the Use of Zinc Oxide in the Rubber Vulcanization Process

**DOI:** 10.3390/toxics10120781

**Published:** 2022-12-13

**Authors:** Cinzia Bragato, Silvia Mostoni, Christian D’Abramo, Maurizio Gualtieri, Francesca Rita Pomilla, Roberto Scotti, Paride Mantecca

**Affiliations:** 1 POLARIS Research Center, Department of Earth and Environmental Sciences, University of Milano-Bicocca, Piazza della Scienza 1, 20126 Milan, Italy; 2 Department of Materials Science (INSTM), University of Milano-Bicocca, Via R. Cozzi 55, 20125 Milan, Italy

**Keywords:** manufactured nanomaterials, nano-safety, in vitro and in vivo models, inflammatory mediators, fish embryo acute toxicity test (FET)

## Abstract

Zinc oxide (ZnO) is the most efficient curing activator employed in the industrial rubber production. However, ZnO and Zn(II) ions are largely recognized as an environmental hazard being toxic to aquatic organisms, especially considering Zn(II) release during tire lifecycle. In this context, aiming at reducing the amount of microcrystalline ZnO, a novel activator was recently synthetized, constituted by ZnO nanoparticles (NPs) anchored to silica NPs (ZnO-NP@SiO_2_-NP). The objective of this work is to define the possible hazards deriving from the use of ZnO-NP@SiO_2_-NP compared to ZnO and SiO_2_ NPs traditionally used in the tire industry. The safety of the novel activators was assessed by in vitro testing, using human lung epithelial (A549) and immune (THP-1) cells, and by the in vivo model zebrafish (*Danio rerio*). The novel manufactured nanomaterial was characterized morphologically and structurally, and its effects evaluated in vitro by the measurement of the cell viability and the release of inflammatory mediators, while in vivo by the Fish Embryo Acute Toxicity (FET) test. Resulting data demonstrated that ZnO-NP@SiO_2_-NP, despite presenting some subtoxic events, exhibits the lack of acute effects both in vitro and in vivo, supporting the safe-by-design development of this novel material for the rubber industry.

## 1. Introduction

Nowadays, rubber is a material used worldwide for many purposes, as tires, gloves, shoes, and many other goods [1], thanks to its unique properties, like hardness, elasticity, and improved elongation at break [2,3,4].

From an industrial perspective, the mechanical properties of rubber are generally improved by adding reinforcing fillers to the rubber matrix, such as SiO_2_, silicates, and nanometric carbon black, which promote the formation of a percolative filler network inside the rubber nanocomposites (NCs) [5,6,7,8,9]. In addition, a curing process, i.e., vulcanization, is performed in order to convert the raw sticky polymer into an elastic material by cross-linking the rubber chains, through poly- to mono-sulfide bridges [10].

Vulcanization rate and efficiency are industrially improved by using ZnO as a curing activator, along with organic accelerators and co-activators (i.e., fatty acids) [11]. Nonetheless, ZnO entails non-negligible potential environmental risks, in particular to aquatic organisms, although potential risk for humans cannot be excluded. Actually, tire wear particles represent a potential hazard for humans and aquatic species [12,13], due to their significant amount in the urban airborne particulate matter (PM) and their impact on aquatic environments as a consequence to leaching phenomena [14], or as micro and nanoplastics species per se [15]. It is worth noting that the exceedance of safe environmental Zn(II) concentration is detected in urban areas [16], thus supporting the need for reducing all the possible sources of Zn ions.

Accordingly, the Environmental Protection Agency (EPA) defined the reduction of ZnO level, a compelling matter in rubber and tire production in addition to in the end-of-life tire recycling treatments [17]. In this context, several candidate materials have been proposed to reduce the amount of the traditional microcrystalline ZnO activator and to improve the efficiency of the curing process at the same time [18,19,20,21]. Recently, a novel activator composed of ZnO nanoparticles (NPs, here after ZnO-NP@SiO_2_-NP) anchored to silica filler NPs was recently proposed, which behave simultaneously as curing activator and filler (i.e., double function filler), allowing a 50% reduction of the ZnO amount currently used in the production of rubber composites for tires [22,23]. 

Since the new ZnO-NP@SiO_2_-NP is made by a core of SiO_2_-NP surface covered by ZnO-NP, it is mandatory to characterize the possible hazards deriving from the use of these novel NPs comparing them to commercial ZnO and SiO_2_ NPs, both alone or mixed together. 

In fact, within the REACH (EC 1907/2006) perspective, every novel manufactured nanomaterial (MNs) has to be tested for address health and safety concerns, also in comparison with traditional or alternative materials used in the process [24]. 

Moreover, the methods to evaluate the MNs potential toxicity following the recent OECD (Organization for Economic Co-operation and Development) guidelines [25,26] and literature approaches [27], which report the test regulations to investigate the human and environmental toxicity of new MNs, involving in vitro and alternative in vivo models, were used [28]. 

In this research paper, the NPs were characterized by transmission electron microscopy (TEM) and dynamic light scattering (DLS) and then the toxicological aspects related to both human and environmental health safety were covered. The in vitro and in vivo investigations were performed using a monoculture of human lung epithelial (A549) and immune (THP-1) cells [12,13] and the zebrafish (*Danio rerio*) model [29]. After NP exposure in vitro, the cytotoxicity and the release of inflammatory mediators IL-8 (Interleukin 8) and RANTES (Regulated on Activation, Normal T cell Expressed and Secreted) were evaluated by ELISA (Enzyme-linked immunosorbent assay) [30,31], while the assessment of nanotoxicity in vivo was performed by the Fish Embryo Acute Toxicity (FET) test [32,33].

The first evidence on the in vitro and in vivo hazard of this new hybrid nano-formulation represents significant information to adopt and possibly develop newly safe-by-design formulation of a curing agent for the rubber industry.

## 2. Materials and Methods

### 2.1. Synthesis of ZnO-NP@SiO_2_-NP

The synthesis of the novel ZnO-NP@SiO_2_-NP was performed according to the procedure reported in [22].

Briefly, 0.3 g of Zn(CH_3_COO)*2H_2_O and 0.28 g of ≥98% purity NaOH, final concentration 0.1 M, (Merck KGaA, Darmstadt, Germany) were dissolved in 70 mL of 99.9% EtOH (Exacta + Optech Labcenter, San Prospero, Italy) at 65 °C; later on, 1.0 g of SiO_2_ NPs (Rhodia Zeosil MP1165 with a surface area of 160 m^2^ g^−1^, Solvay, Bruxelles, Belgium) was added and kept under stirring at 65 °C for 20 min. The resulting ZnO-NP@SiO_2_-NP were filtrated, washed several times with fresh ethanol, and finally dried in oven at 80 °C overnight. The Atomic Emission Spectroscopy (ICP-AES) ZnO content of the novel NPs was 8.0 (±1) wt%, while 92.0 (±1) wt% was of SiO_2_ [22].

### 2.2. Preparation of the Particle Suspensions

The following preparation protocols were used for ZnO-NP@SiO_2_-NPs, commercial ZnO (CAS 1314-13-2) NPs, with a size < 50 nm, purchased from Sigma-Aldrich (Sigma-Aldrich, Milan, Italy) and SiO_2_-NPs. NPs were weighed in a micro-balance (Sartorius, Goettingen, Germany) in sterile conditions, under a laminar flow hood, suspended in sterile ultrapure water. The stock solutions for ZnO-NP@SiO_2_-NPs were 1 mg/mL, while for ZnO and SiO_2_ NPs were 80 µg/mL and 920 µg/mL, respectively.

Successively, ZnO-NP@SiO_2_-NPs were sonicated with a probe-type sonicator until it reached energy 3 kJ/s (Bandelin Sonopuls, Berlin, Germany), in order to obtain a well-dispersed suspension of particles, while ZnO and SiO_2_ NPs were sonicated in an ultrasonic bath (SONICA Soltec, Milano, Italy) for 20 min.

Suspensions were stored at 4 °C temperature, for a period no longer than 30 days. 

### 2.3. Morphological and Surface Characterization

The morphology of ZnO-NP@SiO_2_-NP, ZnO (<50 nm particle size (TEM), Merck KGaA, Darmstadt, Germany) and SiO_2_ NPs was first studied by transmission electron microscopy (TEM). Briefly, 10 µL of NPs suspension (600 µg/mL) in EtOH were dropped on a carbon coated 300-mesh copper grid. Grids were observed with a Jeol JEM 2100Plus (JEOL, Tokyo, Japan) TEM, operating at an acceleration voltage of 200 kV and equipped with an 8 MPx complementary metal oxide semiconductor (CMOS) Gatan Rio9 (Gatan, Pleasanton, CA, USA) digital camera. 

The hydrodynamic size distribution of ZnO-NP@SiO_2_-NP, ZnO and SiO_2_ NPs was evaluated through dynamic light scattering (DLS) analysis by using a Malvern Zetasizer (Malvern WR14 1XZ, UK). The NP hydrodynamic behavior was assessed by dispersing the NPs (final concentration 100 μg/mL) in different media: (i) Milli-Q (mQ) water, (ii) DMEM (Gibco, Life Technologies, Monza, Italy) medium with 1% fetal bovine serum (FBS, Gibco, Life Technologies, Monza, Italy), (iii) Opti-MEM (Gibco, Life Technologies, Monza, Italy) medium supplemented with 1% FBS, and (iv) FET solution (0.1 g NaHCO_3_ and 0.19 g CaSO_4_*2H_2_O; from Sigma-Aldrich, St. Louis, Missouri, MO, USA; 0.1 g instant ocean from Instant Ocean Spectrum Brands, Blacksburg, Virginia, VA, USA). The latter three media (DMEM with 1% FBS, Opti-MEM with 1% FBS and FET solution) were used for A549 cells, THP-1 cells, and for FET experiments, respectively. For the treatment with NPs, the cell culture media were with 1% FBS to reduce, as much as possible, the protein corona [34,35].

### 2.4. Experimental Design

The hazard analyses have been performed on ZnO-NP@SiO_2_-NPs and on bare ZnO-NP and SiO_2_-NP (as representative of the canonical nanoparticles used in tyre curing), alone and mixed. The ZnO-NP@SiO_2_-NP is constituted by ZnO nanoparticles anchored to a silica core particle, at concentrations of 8% and 92%, respectively. Thus, the experiments were conducted comparing the ZnO-NP@SiO_2_-NPs at concentrations of 10, 50, and 100 µg/mL to the corresponding concentrations of ZnO and SiO_2_ nanoparticles, alone or mixed, as reported in Table 1.

In order to compare most precisely the effects of NPs administration in all the experimental groups, which are adherent cell culture (A549), suspension cell culture (THP-1) and zebrafish, the exposure concentrations were calculated as a mass of NPs per surface area. 

### 2.5. In Vitro and In Vivo Hazard Assessment

Hazard assessment was conducted on monoculture of human lung epithelial (A549, ATCC, Manassas, VA, USA) and immune (THP-1 (ATCC) cells, and on the zebrafish (*Danio rerio*) model (Appendix A Table A1). The exposure concentrations of ZnO-NP@SiO_2_-NPs, ZnO-NP, SiO_2_-NP, and ZnO-NP + SiO_2_-NP were tested as reported in Table 1.

A549 cells were seeded using DMEM with 10% of FBS (Gibco, Life Technologies, Monza, Italy) in 6 multiwell plates and kept in an incubator at 37 °C and 5% CO_2_. Cells were treated for 24 h with the different NP concentrations (Table 1) after reaching circa 70% of confluence, and a reference not exposed group (Control Not Treated—Ctrl N.T.) was also considered. The THP-1 were seeded at 1.0 × 10^5^ cells in 12 multiwell plates with Opti-MEM Reduced Serum Medium with 10% FBS added (Gibco, Life Technologies, Monza, Italy). After 24 h, the cells were treated with NPs resuspended in Opti-MEM with 1% FBS (Table 1), and left at 37 °C and 5% of CO_2_ for additional 24 h. At the end of cell exposure, A549 and THP-1 cells were centrifuged for 3′ at 300× *g* and the supernatant tested for their viability. Exposure media were collected and kept at −20 °C until the analyses of the selected inflammatory markers. 

### 2.6. Cell Viability and Inflammatory Markers Release

The A549 cells viability was assessed by the MTT assay. Briefly, cell medium was removed and collected for subsequent analysis, and replaced with a serum-free media and MTT reagent. Cells were then incubated for 3 h at 37 °C. Later, dimethyl sulfoxide (DMSO) was added to cells and, after 15 min of incubation, the plate was analyzed with a TECAN (Infinite 200 PRO series) microplate reader (Tecan Trading AG, Männedorf, Switzerland).

The collected cell medium was used to evaluate IL-8 and RANTES levels by the commercial Enzyme-linked immunosorbent assay (ELISA) kit (Invitrogen, Waltham, MA, USA) according to the manufacturer’s instructions. 

Briefly, cell culture supernatants ware added into 96-well plates coated with monoclonal (anti-human IL-8) or polyclonal (anti-human RANTES antibody) after blocking with 1% bovine serum albumin, followed by incubation with peroxidase-labeled anti-DNA monoclonal or polyclonal antibody. The optical absorbance was measured at 450 nm in an ELISA reader (Tecan Trading AG, Männedorf, Switzerland) and the content of released pro-inflammatory protein, in pg/mL, determined according to the standard curves.

The AlamarBlue test was used to assess cell viability of THP-1 cells after exposure. Briefly, cells were placed in a 1.5 mL vial and centrifuged for 5 min at 300× *g*. The exposure media was recovered and successively used to evaluate the inflammatory response by ELISA assay.

The pellet was resuspended with 900 µL (MW12) of medium with 10% FBS and 100 µL of AlamarBlue. The cells were repositioned to each well and incubated at 37 °C for about 3 h. After incubation, the supernatant was transferred into 96 multiwell plates and then analyzed with TECAN (Infinite 200 PRO series) microplate reader at a wavelength of 570 and 630 nm.

### 2.7. Animal Care

The adult AB wildtype are maintained and bred at the University of Milan-Bicocca zebrafish facility (approved by ATS MetroMilano Prot. n. 0020984—12 February 2018), in a recirculating ZebTec Active Blue aquatic system (Tecniplast, Buguggiate, Italy). All experiments were performed on embryos within 5 days post fertilization (dpf), thus not subject to animal experimentation rules according to European and Italian directives.

### 2.8. Fish Embryo Acute Toxicity Test (FET)

The eco-toxicity of NP suspensions was assessed by the Fish Embryo acute Toxicity test (FET) according to the OECD guidelines. The FET test is a valid alternative animal test for assessing the risk of environmental contaminants for animals and human health [36,37].

Briefly, freshly fertilized zebrafish embryos are exposed to contaminants for a total of 96 h. 

The NPs are suspended in FET solution (0.1 g of NaHCO_3_; 0.1 g of Instant Ocean; 0.19 g of CaSO_4_*2H_2_O for 1 L of solution).

Every 24 h, embryos were screened for lethality, in particular checking coagulation of fertilized eggs, lack of somite formation, lack of detachment of the tail bud from the yolk sac and lack of heartbeat, according to the specific timepoints.

Moreover, sub-lethal endpoints, as reduced yolk resorption, blood congestion, formation of edemata and lack of hatch, were observed from 48 h post fertilization (hpf) to 96 hpf developmental stage [38].

At the end of the exposure period, acute toxicity is determined based on a positive result in one of the four observations, and the LC_50_ (lethal concentration 50) is calculated. 

To assess the EC_50_ (effective concentration 50), which is the concentration of a toxicant that produces a biological response, the sub-lethal endpoints were evaluated.

### 2.9. Statistical Analysis

Data are reported here as means and standard error of the means (SEM) using GraphPad Prism 9.2.0.332 (GraphPad Software). To determine the statistical significance between multiple groups, one-way/two-way ANOVA, followed by Bonferroni’s post hoc test analysis, was employed using GraphPad Prism. The level was considered of significance at <0.05.

## 3. Results

### 3.1. ZnO-NP@SiO_2_-NP Characterization 

**Figure 1:**
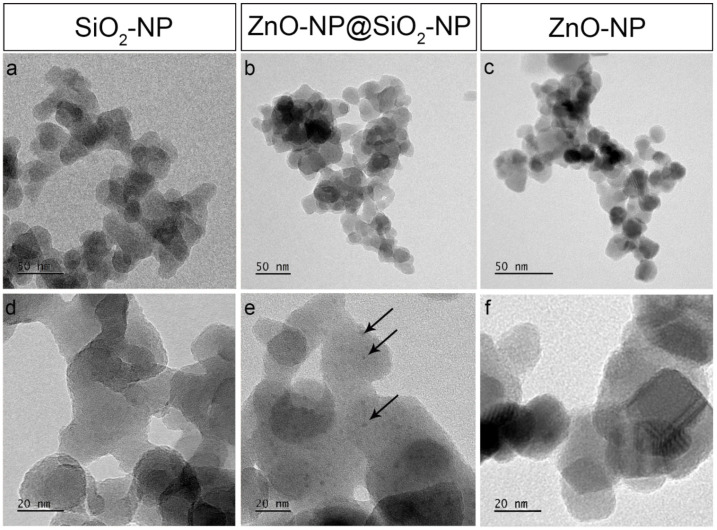
TEM images of SiO_2_ Rhodia (**a**,**d**), ZnO-NP@SiO2-NP (**b**,**e**) and ZnO NPs (**c**,**f**). Arrows in (**e**) point to the black dots covering SiO_2_ NPs corresponding to ZnO NPs.

The ZnO-NP@SiO_2_-NP morphological investigation (TEM images) shows the formation of well distributed nano aggregates of ZnO on the SiO_2_ surface, visible as spots with a size of 4 ± 1 nm (Figure 1b,e). SiO_2_ NPs exhibit an average size of 25 ± 5 nm, with a typical tendency to aggregate forming micro pearls interconnected between each other (Figure 1a,b,d). In addition, the commercial ZnO sample is characterized by almost spherical NPs of 13 ± 2 nm (Figure 1c–f). 

Data from DLS analyses confirm the lack of agglomeration of ZnO-NP@SiO_2_-NP in different media (mQ water, FET solution, DMEM and Opti-MEM with 1% FBS) and at different time points (0 and 2 h) (Table 2). ZnO-NP showed a change in particle hydrodynamic size (possible agglomeration) when suspended in FET solution (921.1 nm) compared to other media (347,833 in mQ water, 395,733 in DMEM and 392.4 in Opti-MEM). 

SiO_2_-NP are characterized by an increase in hydrodynamic diameter after being suspended for 2 h in both cell exposure media, suggesting an ongoing agglomeration process. 

The PdI measurement relative to ZnO-NP@SiO_2_-NP, ZnO-NP, and SiO_2_-NP showed similar values in the different media used, suggesting that the NP suspensions were mid-range monodispersed.

The zeta-potentials, measured in mQ water, of ZnO-NP@SiO_2_-NP (equal to −22.70 mV) and of SiO_2_ NP (equal to −17.50 mV), show that these NPs are negatively charged. On the contrary, zeta-potential of ZnO-NP is +16.87 mV. 

### 3.2. In Vitro Toxicity

The results showed that the cytotoxicity effects after 24 h of treatment with ZnO-NP@SiO_2_-NPs are comparable to those induced by the reference NPs selected.

**Figure 2:**
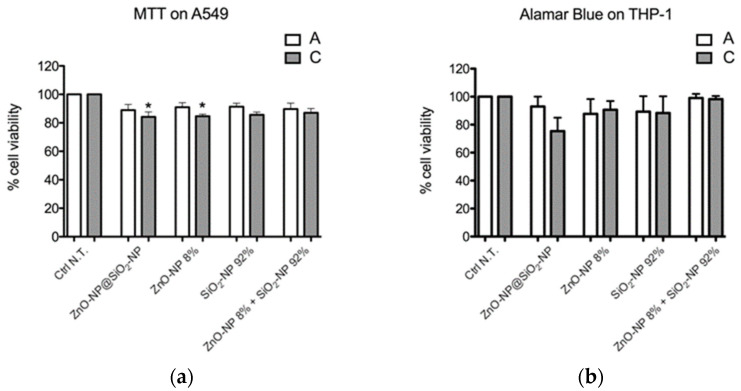
The cell viability was assessed by (**a**) MTT test on A549 cells and (**b**) Alamar Blue on THP-1 cells. Data represent the mean ± SEM of at least three independent experiments. * Statistically different from control sample; * *p* < 0.005. One-way ANOVA + Bonferroni’s test. IC_50_ is reported as >higher concentration tested in both in vitro models. For exposure concentration legend, please refer to Table 1.

In fact, A549 cell viability was reduced only after treatment with the highest concentration (C) of ZnO-NP@SiO_2_-NPs compared to Ctrl N.T. A similar result was obtained with ZnO-NP (concentration C) (Figure 2a).

In THP-1 cells, viability was not significantly decreased after NP treatments at both concentrations tested (Figure 2b). 

**Figure A1:**
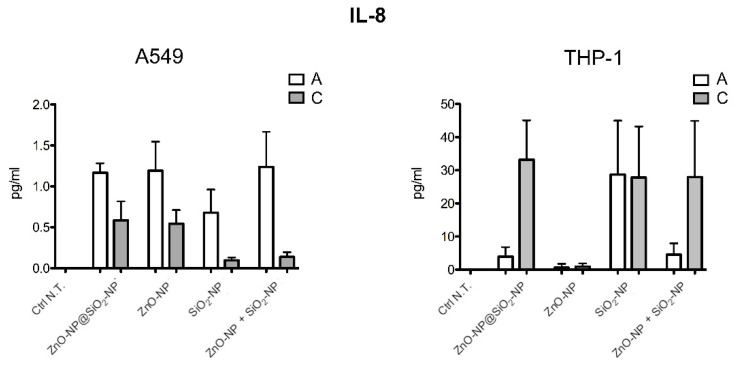
IL-8 levels on A549 cells (**left panel**) and on THP-1 cells (**right panel**). Data represent the mean ± SEM of at least three independent experiments. For exposure concentration legend, please refer to the Table 1.

The inflammatory mediator’s analysis (Figure A1) showed non-significant effects in A549 cells (Figure A1 left panel) and THP-1 cells (Figure A1 right panel), although this latter model showed a tendency to increased IL-8 after treatment with ZnO-NP@SiO_2_-NP, SiO_2_-NP, and ZnO-NP + SiO_2_-NP at the highest concentration (C).

**Figure A2:**
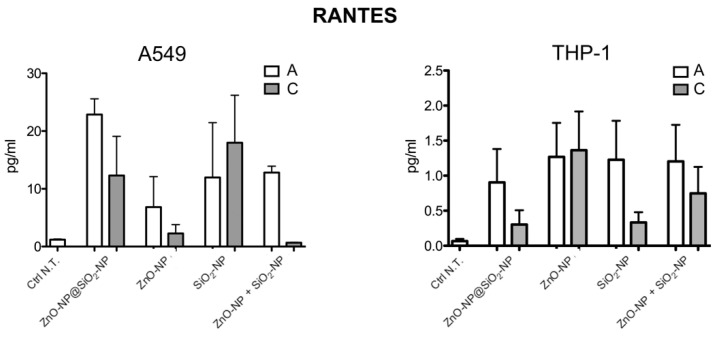
RANTES levels on A549 cells (**left panel**) and on THP-1 cells (**right panel**). Data represent the mean ± SEM of four independent experiments. For exposure concentration legend, please refer to the Table 1.

RANTES levels (Figure A2, left panel for A549 and right panel for THP-1) were not statistically significant in the four exposure conditions and for both of the in vitro models.

### 3.3. Aquatic Toxicity on Zebrafish Embryos—FET Test

After fertilization, zebrafish embryos were exposed to NPs at the concentrations A, B, and C. For this project, the total embryos observed were N = 120 (ZnO-NP@SiO_2_-NP), N = 120 (ZnO-NP), N = 120 (SiO_2_-NP), and N = 120 (ZnO-NP + SiO_2_-NP) pertaining to 5 different experiments.

**Figure 3:**
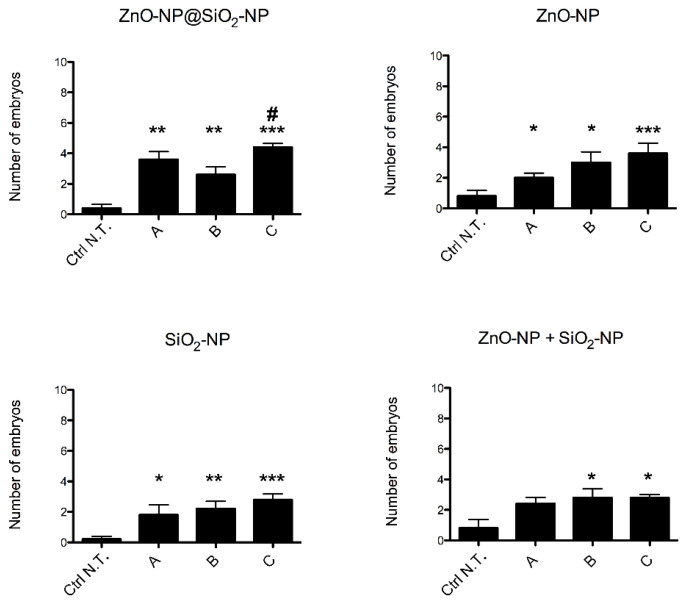
Mortality of zebrafish embryos after exposure to ZnO-NP@SiO_2_-NP, ZnO-NP, SiO_2_-NP, and ZnO-NP + SiO_2_-NP NPs suspensions at 96 hpf. The NP concentrations administered are A, B, and C. The results are presented as a mean ± SEM of five independent experiments. * *p* < 0.05, ** *p* < 0.01, *** *p* < 0.001, with respect to Ctrl N.T.; # *p* < 0.05 with respect to both ZnO-NP@SiO_2_-NP concentration B and C. One-way ANOVA with post hoc Bonferroni’s test. For exposure concentration legend, please refer to Table 1. Analyses were performed on a total of n = 120 embryos for each experimental condition, collected during five independent experiments.

Embryos mortality rate (Figure 3) observed after exposure with ZnO-NP@SiO_2_-NP, ZnO-NP, SiO_2_-NP and ZnO-NP + SiO_2_-NP is below 20%, although the increases are statistically significant when compared to the control group. Nonetheless, given the low rate of mortality, the LC_50_ is to be considered much higher than the highest concentration (C) tested for each NP (alone or in combination). 

**Figure 4:**
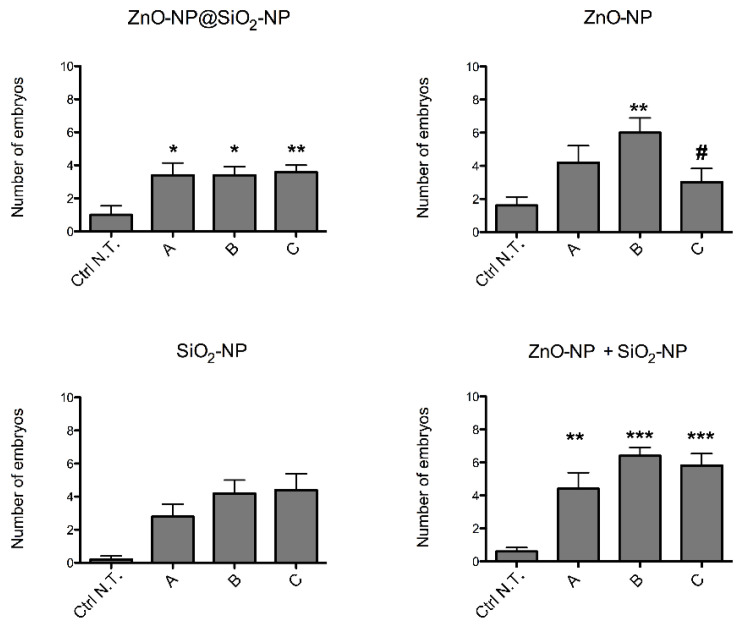
Sublethal defects observed in zebrafish embryos after exposure to ZnO-NP@SiO_2_-NP, ZnO-NP, SiO_2_-NP, and ZnO-NP + SiO_2_-NP NPs suspensions at 96 hpf. The NPs concentrations administered are A, B, and C. The results are presented as mean ± SEM of five independent experiments. * *p* < 0.05, ** *p* < 0.01, *** *p* < 0.001, with respect to Ctrl N.T.; # *p* < 0.05 with respect to both ZnO-NP@SiO_2_-NP at concentrations B and C. One-way ANOVA with post hoc Bonferroni’s test. For exposure concentration legend, please refer to Table 1. Analyses were performed on a total of n = 120 embryos for each experimental condition, collected during five independent experiments.

The total number of embryos with sublethal endpoints observed after exposure (Figure 4) show minor, but significant increases with all the NPs. Interestingly, the average number of malformed embryos per concentration of exposure with the novel NPs is lower than the outcomes observed with the combined exposure of SiO_2_ and ZnO NPs. In addition, SiO_2_ NPs average malformation were not significant, but the co-exposure of ZnO and SiO_2_ NPs seems to have higher effects than SiO_2_ or ZnO alone. Given the low numbers of average malformed embryos, the EC_50_ of the ZnO-NP@SiO_2_-NP was not calculated, and it has to be assumed higher than the highest concentration (C) of exposure used in our experiments. 

**Figure 5:**
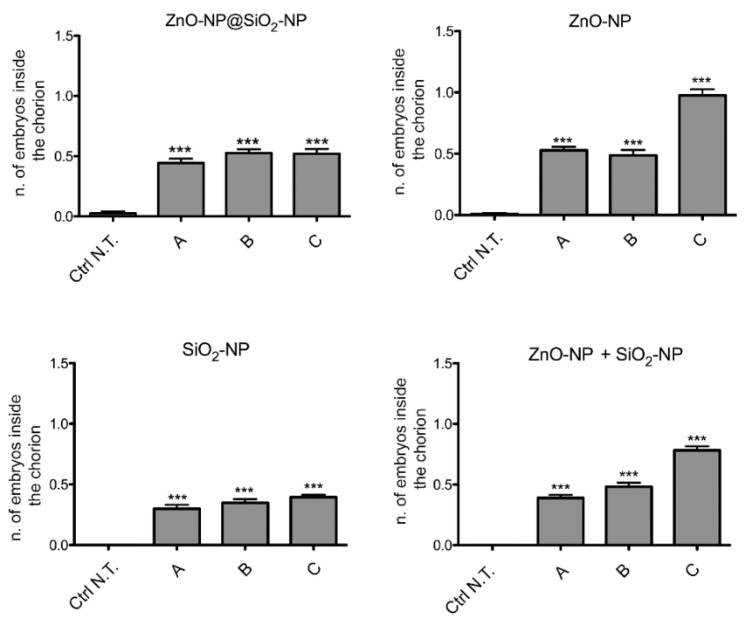
Effects of ZnO-NP@SiO_2_-NP, ZnO-NP, SiO_2_-NP, and ZnO-NP + SiO_2_-NP NPs on embryos hatching. The graphs show the embryos that were alive and almost inside the chorion at 96 hpf. *** *p* < 0.001, with respect to Ctrl N.T. One-way ANOVA with post hoc Bonferroni’s test. For exposure concentration legend, please refer to Table 1. Analyses were performed on a total of n = 120 embryos for each experimental condition, collected during five independent experiments.

Among the sublethal endpoints, the lack of hatch is the more significant defect observed after the administration of ZnO-NP@SiO_2_-NP, ZnO-NP, SiO_2_-NP, and ZnO-NP + SiO_2_-NP (Figure 5). 

**Figure 6:**
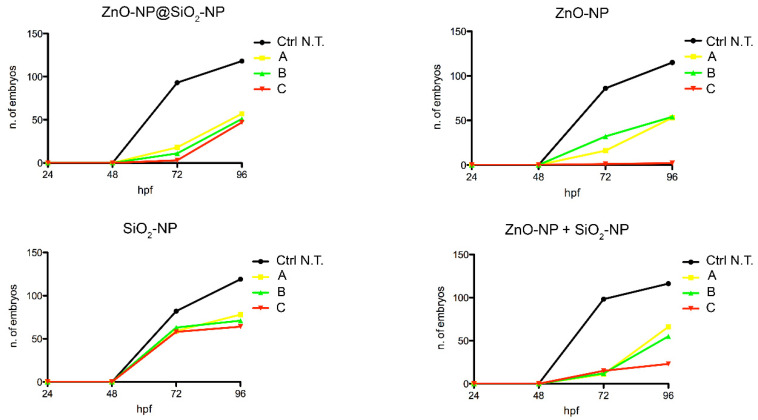
Hatching time curves of 96 hpf embryo non exposed or exposed to ZnO-NP@SiO_2_-NP, ZnO-NP, SiO_2_-NP, and ZnO-NP + SiO_2_-NP NPs. The delay is visible after NP administration, in particular after exposure to ZnO-NP and ZnO-NP + SiO_2_-NP at the higher concentration. For exposure concentration legend, please refer to Table 1. Analyses were performed on a total of n = 120 embryos for each experimental condition, collected during five independent experiments.

In this case, the number of embryos that are unable to exit the chorion after exposure to the NPs suspension is concentration-dependent, and more evident after ZnO-NP and ZnO-NP + SiO_2_-NP treatments. Interestingly, comparing the highest concentration of exposure among the different NPs, it is evident that bare ZnO-NPs have a higher effect on the lack of hatch defect, while the same total amount of ZnO, but linked to the silica core, is not inducing a similar increase of defects. This is confirmed by comparing the hatching time curves (Figure 6). For the ZnO-NP@SiO_2_-NP, a recovery of hatching at all the concentrations tested is evident after 72 h of exposure. Hatching time resulted in not being affected by SiO_2_-NPs. In fact, the start of hatching time is retained at 48 h, even if the number of hatched embryos is lower. The ZnO-NPs instead, alone or in combination with SiO_2_, are able to perturb the hatching time more drastically, with the highest ZnO-NP concentration that blocked the hatching until the end of the exposure period.

## 4. Discussion

Object of this research is to evaluate the hazard to human beings and the environment of ZnO-NP@SiO_2_-NP, a new nanomaterial synthetized and produced with the intent of reducing the amount of microcrystalline ZnO activator used in rubber processes. 

The results observed after the structural analyses of NPs used showed that ZnO-NP@SiO_2_-NP did not exhibit relevant changes in the hydrodynamic diameter in water-based suspensions. In fact, an ongoing agglomeration process was observed only in SiO_2_-NPs suspended in both cell media used. The motivation could be related to the “protein corona” effect, caused by the NP interaction with various biomolecules, present in cells media, to form a corona. We hypothesize that this effect is visible only in the bare amorphous SiO_2_-NPs, but not in ZnO-NP@SiO_2_-NP, because, in the latter, the presence of ZnO nanoparticles, attached to the underlying silica core, actually modify the surface characteristics of the silica particles. This is can be related also to the lower zeta-potential of the novel NPs, compared to the naked silica NPs [36,37].

Regarding the different z-potential observed, which is negative for ZnO-NP@SiO_2_-NP and SiO_2_-NPs, but not for ZnO-NP, we speculate that the reason could be justified considering that the structure of ZnO-NP@SiO_2_-NP is mainly constituted by silica (Figure 1e) and that it is synthesized in a basic environment that leaves a predominant surface negative charge. Similar results have been reported for silica nanoparticles doped with metal and transition elements with positive charge [39,40].

In the framework of this study, the use of these novel NPs may lead to an actual reduction in the total mass of ZnO used in the rubber tire production [22]. Therefore, reducing the expected exposure for humans and the environment will determine an overall reduction of the risk from tire wear related emissions. 

In order to evaluate the hypothetical exposure effects of ZnO-NP@SiO_2_-NP on human beings, we used A549 and THP1-1 cellular models. We tested the cell viability, as the first acute toxic effect observation, and the IL-8 and RANTES levels. IL-8 and RANTES are important pro-inflammatory chemokines characterized by an early involvement in the inflammatory response. The first recruits neutrophils to the site of damage [30], while the latter attracts and activates leukocytes [31].

The results obtained showed that the novel ZnO-NP@SiO_2_-NPs present a hazard profile comparable, if not lower, than the ZnO-NP, SiO_2_-NP, and ZnO-NP + SiO_2_-NP. 

In fact, in the cellular models, the ZnO-NP@SiO_2_-NP revealed low cytotoxic effects, with only minor reduction at the higher concentration tested. Reduction of cytotoxic effects has also been related to the zeta-potential of the NPs [40]. Shahabi and colleagues reported that the positively charged NPs could be internalized into cells more easily than negative ones. Thus, the specific zeta-potential of the novel nanoparticles may be related to the negligible effects on cells, although more specific tests will be required. 

Similarly, the inflammatory responses were low and non-significant after 24 h of exposure. Low toxicity and low inflammatory profile of novel NMs are essential for their safety. The concentrations of ZnO NPs generally used in the literature are within the range of 25 to 100 μg/mL, and the effects reported in vitro almost suggest safety for this NP [41]. The same is reported for toxicity of SiO_2_ NPs that occurs to be dependent on dose, size, time, and temperature in addition to on the tissue-specificity. Furthermore, concentrations greater than 100 μg/mL are needed to achieve 24 h EC_50_ values in in vitro models [42,43]. 

The potential adverse effects that ZnO-NP@SiO_2_-NPs can have on the environment were evaluated using the zebrafish model and the FET test.

Zebrafish share many gene functions with mammals, making this model a useful system for studying toxic effects of different materials and predict what potentially could happen to human beings [38]. Moreover, some biological endpoints derived from FET could be helpful in understanding the NP toxicity, and the zebrafish embryos could be a predictive model for the risk assessment of NP toxicity [39].

Ecotoxicity outcomes for the composite NPs, while showing a somewhat higher embryotoxic effect (Figure 3), clearly show lower sublethal effects. The embryotoxicity outcomes here reported are in line ZnO NP effects previously investigated and mostly associated with zinc ions release and with the interactions of particle/aggregates with the embryos chorion [44], while, for SiO_2_, the hazard outcome is related mainly to the size of NPs used for testing [45].

Additional analyses are, however, required to properly understand this higher embryo toxic effect of the composite nanomaterial at the higher concentration used (100 μg/mL, Figure 5).

In fact, we can hypothesize that some adverse effects observed (i.e., higher mortality rate) may be due to the increased reactivity of core-shell ZnO-NP@SiO_2_-NP based on its novel structure, more than to the relative quantity of ZnO and SiO_2_ elements. In fact, while the ZnO used as comparison for our experiments has a nominal diameter of <50 nanometers (nm), the novel NPs are composed by ZnO particles with a nominal diameter of only 4 to 10 nm. This extreme difference in the ZnO particle dimension might promote specific and unexpected membrane–particles interaction. It is worth noting, in the research work from Kim and colleagues [46], that the ZnO-NPs concentrations found in the surface water environmental medium were in the range 1–60 μg/L (data for sediment, sewage, sludge, soil, and wastewater are 0.49–56, 13.6–64.7, 0.026–0.66, and 0.22–1.42 μg/L, respectively). Based on these results, the theoretical LC_50_ here reported is however hundreds of times higher than the expected environmental concentration in freshwater, suggesting the absence of an environmental hazard for the new hybrid NPs. 

Interestingly for environmental safety, the embryos presenting a lack of hatch have a lower rate after ZnO-NP@SiO_2_-NP administration, compared to the results obtained after the exposure to ZnO-NP, SiO_2_-NP and ZnO-NP + SiO_2_-NP nanoparticles.

Even the sublethal defects observed are reduced after ZnO-NP@SiO_2_-NP exposure, if compared to the other NPs examined. 

It is of interest that here we report clear differences in the time of hatching curves between the novel NPs and the reference ZnO-NP. 

It is known that normally the hatching period occurs from 48 hpf to 72 hpf [47], but whenever some perturbation arises, a delay in embryos exiting from chorion is detectable, compared to control embryos. The chorion envelops the embryos and is considered a barrier to the entry of NPs into the embryos. It is a semi-permeable structure, characterized by pore canals with a size of approximately 0.6–0.7 μm. These pores are larger than the size of NPs, and the effect of the chorion on NP transport and subsequent biological toxicity may be analyzed in the future, in particular when NPs agglomerate or interact with chorion surface proteins [48].

Many factors can be the cause of hatching delay in zebrafish embryos, as a change in the surface mechanical properties of chorion induced by the direct adherence/adsorption of NPs aggregates. The NPs that aggregate and adhere to chorion can interfere with the digestive function of the chorionic hatching enzyme as well, or can cause a depletion of oxygen exchange resulting in hypoxia, in addition to excessive production of reactive oxygen species (ROS) [49].

## 5. Conclusions

In conclusion, the novel ZnO-NP@SiO_2_-NP can be considered a relatively safe nanomaterial which combines the improved efficiency during the vulcanization process of ZnO to a relative reduction of its hazard, above all when considering the aquatic environment. 

Significantly, these new NPs require a lower amount to properly function as curing agent and, therefore, if applied in substitution for the classical ZnO-NPs, will likely determine in the future a significant reduction of Zn ions release from tire wears and, in turn, the adverse environmental effect related to these metal ions. 

Moreover, some of the ZnO-NP@SiO_2_-NP characteristics, as the hydrodynamic diameter stability in water-based suspensions, make this novel manufactured nanomaterial attractive for a variety of applications. 

However, to improve the knowledge on the hazards for human and aquatic health, future studies may gain advantages from investigating the chronic effects over prolonged exposure periods, comparing the effects of NMs released from tires produced with traditional and new technologies.

## Figures and Tables

**Table 1:** Nanoparticles’ concentrations used in the experiments performed. The different concentrations of exposure are identified with capital letters with “A” referring always to the lowest concentration used, whatever the particles, “C” representing the highest concentration and “B” (used only for Zebrafish experiments) the concentration in between. In the following figures, these letters will be used instead of the whole description of exposure concentration.

***ZnO-NP@SiO_2_-NPs Concentrations Used***	
**A**	10 µg/mL of ZnO-NP@SiO_2_-NP (equivalent to 0.8 µg/mL of ZnO and 9.2 µg/mL of SiO_2_)
**B**	50 µg/mL of ZnO-NP@SiO_2_-NP—only in Zebrafish
**C**	100 µg/mL of ZnO-NP@SiO_2_-NP (equivalent to 8 µg/mL of ZnO and 92 µg/mL of SiO_2_)
***ZnO-NP Concentrations Used***	
**A**	0.8 µg/mL of ZnO
**B**	4 µg/mL of ZnO—only in Zebrafish
**C**	8 µg/mL of ZnO
***SiO_2_-NPs Concentrations Used***	
**A**	9.2 µg/mL of SiO_2_
**B**	46 µg/mL of SiO_2_—only in Zebrafish
**C**	92 µg/mL of SiO_2_
***ZnO-NP + SiO_2_-NPs Concentrations Used***	
**A**	10 µg/mL of ZnO-NP + SiO_2_-NP (equivalent to 0.8 µg/mL of ZnO + 9.2 µg/mL of SiO_2_)
**B**	50 µg/mL of ZnO-NP + SiO_2_-NP—only in Zebrafish (equivalent to 4 µg/mL of ZnO + 46 µg/mL of SiO_2_)
**C**	100 µg/mL of ZnO-NP + SiO_2_-NP (equivalent to 8 µg/mL of ZnO + 92 µg/mL of SiO_2_)

**Table 2:** Z-average (nm) and polydispersity index (PdI) of 100 µg/mL ZnO-NP@SiO_2_-NP, ZnO-NP, and SiO_2_-NP measured by dynamic light scattering (DLS) and in different media (mQ water, FET solution, DMEM with 1% FBS, and Opti-MEM with 1% FBS culture media). Means ± SD of three replicates.

NPs (100 µg/mL)	Medium	Time (h)	z-Average (nm) ± SD	PdI ± SD
ZnO-NP@SiO_2_-NP	mQ	0	331 ± 8	0.51 ± 0.08
	FET	0	330 ± 20	0.49 ± 0.04
	DMEM 1% FBS	0	258 ± 1	0.48 ± 0.01
		2	330 ± 30	0.54 ± 0.05
	Opti-MEM 1% FBS	0	270 ± 10	0.47 ± 0.07
		2	240 ± 6	0.42 ± 0.02
ZnO-NP	mQ	0	350 ± 30	0.58 ± 0.04
	FET	0	920 ± 40	0.5 ± 0.2
	DMEM 1% FBS	0	400 ± 40	0.57 ± 0.03
		2	316 ± 4	0.41 ± 0.05
	Opti-MEM 1% FBS	0	390 ± 10	0.56 ± 0.03
		2	384 ± 6	0.56 ± 0.02
SiO_2_-NP	mQ	0	229 ± 4	0.214 ± 0.002
	FET	0	225 ± 3	0.214 ± 0.002
	DMEM 1% FBS	0	730 ± 20	0.54 ± 0.04
		2	2030 ± 30	0.46 ± 0.04
	Opti-MEM 1% FBS	0	420 ± 20	0.294 ± 0.006
		2	1150 ± 30	0.63 ± 0.09

## Data Availability

Not applicable.

## Appendix A

**Table A1:** List of selected papers reporting the use of biological material to analyze the effects of nanoparticles (from 2019).

Nanoparticles	Biological Material	Reference
Organic and inorganic nanoparticles (NPs)	Adult Stem Cells (ASCs)	[50]
Iron oxide nanoparticles (IONPs)	Primary cultures of brain cells	[51]
Various types of nanoparticles	Cardiac stem cells	[52]
Gold nanoparticles	breast cancer SK-BR-3 cells	[53]
titanium dioxide (TiO_2_) nanoparticles	A549 cells	[54]
ZnO nanoparticles	Caco-2 cells	[41]
biosynthesized silver nanoparticles (ScAgNPs)	THP-1 cells	[55]
Cerium dioxide nanoparticles (CeO_2_ NPs)	A549 cells and THP-1 cells (co-culture)	[56]
Silver (Ag), titanium dioxide (TiO_2_), and zinc oxide (ZnO) nanoparticles	THP-1 cells	[57]
Tantalum (Ta) and Titanium (TiO_2_) NPs	THP-1 cells	[58]
Silver nanoparticle (AgNP)	THP-1 cells	[59]
Polystyrene NP (PLNP)	A549 cells and THP-1 cells	[60]
Amino-functionalized silicon nanoparticle (NH2SiNP)	MC3T3-E1 and PC12 cells and *Danio rerio* (embryos)	[61]
Iron nanoparticles	*Xenopus laevis* (embryos)	[62]
Titanium dioxide NPs (n-TiO(2))	*Danio rerio* (embryos)	[63]
Silicon dioxide nanoparticles (nano-SiO(2))	*Danio rerio* (embryos)	[64]
Ag nanoparticles (NPs), CuO NPs, silica NPs, polymeric NPs, quantum dots	*Danio rerio* (embryos)	[65]
Zirconia oxide nanoparticles (ZrO_2_NPs)	*Danio rerio* (embryos)	[66]
Polyethylene glycol (PEG)-modified SiNPs	*Danio rerio* (embryos)	[67]
Silver (AgNPs), copper nanoparticles (CuNPs)	*Danio rerio* (embryos)	[68]
Silver nanoparticles (Ag NPs) of three different sizes (10, 40, and 100 nm)	*Danio rerio* (embryos)	[69]
Hollow selenium nanoparticles (hSeNPs)	*Danio rerio* (embryos)	[70]
Copper oxide nanoparticles (CuO NPs)	*Danio rerio* (embryos)	[71]
Synthetic silver nanoparticles (AgNPs)	*Danio rerio* (embryos)	[72]
Fluorescently-labeled SiO_2_ NPs of 25 and 115 nm	*Danio rerio* (embryos)	[73]
Citrate-functionalized IONPs (γ-Fe_2_O_3_) NPs	*Danio rerio* (embryos)	[74]
Silver nanoparticles (AgNPs)	*Danio rerio* (embryos)	[48]
Metal nanoparticles (Au, Ag, Cu), and metal oxide nanoparticles (TiO_2_, Al_2_ O_3_, CuO, NiO, ZnO)	*Danio rerio* (embryos)	[75]
Zinc oxide nanoparticles (ZnONPs)	*Danio rerio* (embryos)	[76]
Sliver nanoparticles (AgNPs)	*Danio rerio* (embryos)	[77]
Zinc oxide nanoparticles (ZnONPs)	Marine Crustaceans	[78]
